# Extracellular Vesicles From High Glucose-Treated Podocytes Induce Apoptosis of Proximal Tubular Epithelial Cells

**DOI:** 10.3389/fphys.2020.579296

**Published:** 2020-11-02

**Authors:** Ying Huang, Ruizhao Li, Li Zhang, Yuanhan Chen, Wei Dong, Xingchen Zhao, Huan Yang, Shu Zhang, Zhiyong Xie, Zhiming Ye, Weidong Wang, Chunling Li, Zhilian Li, Shuangxin Liu, Zheng Dong, Xueqing Yu, Xinling Liang

**Affiliations:** ^1^The Second School of Clinical Medicine, Southern Medical University, Guangzhou, China; ^2^Division of Nephrology, Guangdong Provincial People’s Hospital, Guangdong Academy of Medical Sciences, Guangzhou, China; ^3^Institute of Hypertension, Zhongshan School of Medicine, Sun Yat-sen University, Guangzhou, China; ^4^Department of Cellular Biology and Anatomy, Medical College of Georgia at Augusta University and Charlie Norwood Veterans Affairs Medical Center, Augusta, Georgia

**Keywords:** podocytes, proximal tubular epithelial cells, diabetic kidney disease, microRNA, extracellular vesicles

## Abstract

Diabetic kidney disease (DKD) is a serious and common complication of diabetes. Extracellular vesicles (EVs) have emerged as crucial vectors in cell-to-cell communication during the development of DKD. EVs may mediate intercellular communication between podocytes and proximal tubules. In this study, EVs were isolated from podocyte culture supernatants under high glucose (HG), normal glucose (NG), and iso-osmolality conditions, and then co-cultured with proximal tubular epithelial cells (PTECs). MicroRNAs (miRNA) sequencing was conducted to identify differentially expressed miRNAs of podocyte EVs and bioinformatics analysis was performed to explore their potential functions. The results showed that EVs secreted from HG-treated podocytes induced apoptosis of PTECs. Moreover, five differentially expressed miRNAs in response to HG condition were identified. Functional enrichment analysis revealed that these five miRNAs are likely involved in biological processes and pathways related to the pathogenesis of DKD. Overall, these findings demonstrate the pro-apoptotic effects of EVs from HG-treated podocytes on PTECs and provide new insights into the pathologic mechanisms underlying DKD.

## Introduction

Diabetic kidney disease (DKD) is a serious and common microvascular complication of diabetes mellitus (DM) and a primary cause of end-stage renal disease (ESRD; Tuttle et al., 2014). Although current clinical therapies and interventions can attenuate the severity of DKD, at present, there is no curative treatment as the mechanisms underlying DKD have not yet been fully described.

Podocyte injury is considered an early and crucial event in the onset and development of DKD (Li et al., 2007). Podocytes are highly differentiated cells lining the outer surface of the glomerular basement membrane that are important for the maintenance of the structure, function, and integrity of the glomerular filtration barrier. Previous studies conducted by our team and others have shown dysfunction and depletion of podocytes under diabetic pathogenic conditions, such as hyperglycemia, increased production of glycation end-products, oxidative stress, and inflammation (Dai et al., 2017; Li et al., 2018; Zhao et al., 2018). The dysfunction and depletion of podocytes causes proteinuria (Mundel and Shankland, 2002), which is an intensive instigator of tubulointerstitial inflammation and fibrosis (Wang et al., 2000; Abbate et al., 2006; Gorriz and Martinez-Castelao, 2012). Filtered proteins induce the production of cytokines and chemokines by proximal tubular epithelial cells (PTECs), which subsequently leads to the infiltration of inflammatory cells and, ultimately, to tubulointerstitial fibrogenesis and progression to ESRD. Tubulointerstitial fibrosis is reportedly closely related to declining renal function (Nath, 1992; Taft et al., 1994). However, a number of diabetic patients progress to ESRD in the absence of proteinuria (Piscitelli et al., 2017; Viazzi et al., 2017). In addition, kidney biopsies of some DKD patients with normal albuminuria/microalbuminuria, but elevated serum creatinine, revealed severe tubular interstitial lesions with mild glomerular lesions. Therefore, apart from proteinuria, there may exist other mechanisms that mediate the crosstalk between podocytes and the proximal tubules.

Extracellular vesicles (EVs) are small membranous vesicles that released into the extracellular space by various cell types under both physiological and pathological conditions. EVs include two major categories: exosomes ranging in diameter from 30 to 150 nm and microparticles ranging in diameter from 50 to 1,000 nm (Tkach and Théry, 2016; van Niel et al., 2018). EVs have emerged as novel and crucial vectors in cell-to-cell communication *via* the delivery of bioactive molecules, which include proteins, lipids, mRNA, and MicroRNAs (miRNA), originating from parental cells (EL Andaloussi et al., 2013). Increasing evidence has revealed that EV-mediated intercellular communication plays important roles in the development of DKD. Exosomes derived from high glucose (HG)-treated glomerular endothelial cells (Wu et al., 2016, 2017), glomerular mesangial cells (Wang et al., 2018), and macrophages (Zhu et al., 2019) transfer injury information to neighboring cells, which accelerates the development of DKD. Structurally, podocytes are located on the outer surface of the glomerular capillary loops adjacent to the proximal tubules. Therefore, the proximal tubule is a likely site for the interactions of podocyte EVs (Lv et al., 2019). Also, studies have showed that EVs from podocytes can interact with PTECs and induce pro-fibrotic or apoptotic responses (Munkonda et al., 2018; Jeon et al., 2020). Thus, we proposed that under HG condition, EVs from podocytes can be internalized and exert harmful effects on PTECs.

miRNAs are small and non-coding RNAs that regulate gene expression through the repression of translation or degradation of mRNAs (Bartel, 2004). Previous evidence has demonstrated the key role of miRNA in DKD pathogenesis (Kato and Natarajan, 2015; Lu et al., 2017). EV miRNAs are emerging as critical factors involved in the pathogenesis of many diseases (Valadi et al., 2007; Zhang et al., 2015), suggesting that EV miRNAs may be novel molecular mediators of the development of DKD. The aim of the present study was to determine whether EVs derived from podocytes can be internalized and induce apoptosis of PTECs *in vitro*. Also, miRNA sequencing was performed to identify differentially expressed miRNAs in podocytes EVs in response to HG condition. In addition, bioinformatics analysis was conducted in order to explore the potential biological functions of EVs from HG-treated podocytes.

## Materials and Methods

### Cell Culture and Treatment

Conditionally immortalized mouse podocytes and immortalized mouse PTECs were cultured as described previously (Li et al., 2018; Zhang et al., 2019). Podocytes were grown in Roswell Park Memorial Institute 1640 medium (Gibco BRL) supplemented with 10% fetal bovine serum (FBS; Gibco BRL) and 50 U/ml of interferon-γ (ProSpec) under an atmosphere of 5% CO_2_/95% air at 33°C. Once confluence reached 70–80%, the podocytes were subcultured at 37°C in Dulbecco’s modified Eagle’s medium (DMEM; Gibco BRL) supplemented with 5% FBS on collagen-coated dishes for 10 days in the absence of interferon-γ to promote differentiation. Following differentiation, the podocytes were grown in serum-free DMEM for 24 h to induce quiescence. Afterward, the podocytes were cultured in DMEM supplemented with 2% exosome-depleted FBS (System Biosciences) and divided into three groups: a normal glucose group (NG-podo, 5.3 mM glucose), high-glucose group (HG-podo, 30 mM glucose), and mannitol group (MA-podo, 5.3 mM glucose + 24.7 mM mannitol; osmolality control). After 48 h of continuous treatment, the cell culture supernatants were collected for EV isolation. Mouse PTECs were cultured in DMEM/F12 (Gibco BRL) supplemented with 10% FBS under an atmosphere of 5% CO_2_/95% air at 37°C and passaged every 3 days. PTECs were cultured in serum-free DMEM/F12 for 12 h to induce quiescence before being dividing into two groups: a normal glucose group (NG-PTECs) and high-glucose group (HG-PTECs). Each group was treated with podocyte EVs for 48 h.

### EV Isolation

EVs from the supernatants of podocyte cultures were isolated using ExoQuick-TC Exosome Precipitation Solution (System Biosciences) in accordance with the manufacturer’s recommendations. In brief, cell culture supernatants were centrifuged at 3000 × *g* for 15 min to remove cells and cellular debris. Then, the resulting supernatants were collected. Following the addition of a 20% volume of ExoQuick-TC precipitation solution, the supernatants were incubated overnight at 4°C and then centrifuged at 1500 × *g* for 30 min to pellet the EVs. The EVs were resuspended in 1× sterile phosphate-buffered saline (PBS) and stored at −80°C until use.

### Transmission Electron Microscopy

Following negative staining, transmission electron microscopy (TEM) was used to reveal the morphology of the isolated EVs. The samples were dropped onto a formvar/carbon-coated copper grid for 5 min and then stained with 4% uranyl acetate for 10 min. After drying at room temperature, EVs on the grid were imaged under a transmission electron microscope (JEM-1400 PLUS; JEOL).

### Nanoparticle Tracking Analysis

Nanoparticle tracking analysis (NTA) was performed to evaluate the size distribution of the isolated EVs with the use of a NanoSight NS300 instrument (Malvern Panalytical) and the corresponding analytical software NTA version 3.3. The EVs suspension was diluted in 1 × PBS and the movement of vesicles was measured three times for a duration of 30 s each.

### Western Blot Analysis

Western blot analysis was performed to detect the positive protein markers of EVs. EVs from the same cell number were used for western blotting quantification. EV samples were lysed with radioimmunoprecipitation assay buffer (Beyotime). EV proteins were separated by 9% sodium dodecyl sulfate-polyacrylamide gel electrophoresis and then transferred onto polyvinylidene fluoride membranes (EMD Millipore). After blocking with 5% non-fat milk for 1 h at room temperature, the membranes were incubated overnight at 4°C with the following primary antibodies:anti-CD63 (ab217345; dilution, 1:1,000; rabbit immunoglobulin [Ig]G; Abcam), anti-CD9 (ab92726; dilution, 1:1,000; rabbit IgG; Abcam), anti-Alix (92880; dilution, 1:1,000; rabbit IgG; Cell Signaling Technology), and anti-calnexin (ab22595; dilution, 1:1,000; rabbit IgG; Abcam). Afterward, the membranes were incubated with horseradish peroxidase-conjugated goat anti-rabbit IgG (7074; dilution, 1:3,000; Cell Signaling Technology) for 1 h at room temperature. Finally, the protein signals were detected by enhanced chemiluminescence (EMD Millipore) and visualized with an ImageQuant LAS500 instrument (GE Healthcare Bio-Sciences).

### EV Uptake Experiment

EVs were labeled using the PKH67 Green Fluorescent Cell Linker Kit (Sigma-Aldrich) in accordance with the manufacturer’s instructions. After incubation for 48 h with the labeled EVs, the PTECs were fixed with 4% paraformaldehyde for 30 min, then permeabilized with 0.5% Triton X-100 for 10 min, and finally stained with 4′,6-diamidino-2-phenylindole (DAPI, Sigma-Aldrich) for 10 min. The resulting fluorescent signals were observed under a confocal laser microscope (TCS-SP5; Leica Microsystems GmbH).

### Flow Cytometry

Apoptotic PTECs were examined by flow cytometry using an Annexin V-FITC/propidium iodide (PI) apoptosis detection kit (Nanjing KeyGEN), as previously described (Zhang et al., 2019). Briefly, after treatment, PTECs were collected, resuspended in 500 μl of 1×binding buffer, and then stained with 5 μl of Annexin V-FITC and 5 μl of PI. Cell fluorescence was then analyzed using a BD FACSVerse™ flow cytometer (BD Biosciences).

### TUNEL Staining

TUNEL staining was performed using a One-step TUNEL cell apoptosis detection kit (Nanjing KeyGEN) following the manufacturer’s instructions. Cells were fixed with 4% paraformaldehyde for 30 min and permeabilized with 1% Triton X-100 for 5 min. Cell samples were then incubated with terminal deoxy-nucleotidyl transferase (TdT) enzyme reaction mixture at 37°C for 1 h, followed by incubated with streptavidin-fluorescein labeling buffer at 37°C for 30 min. Nuclei were stained with DAPI for 5 min at room temperature. The number of TUNEL-positive cells was counted under a confocal laser microscope (Nikon C2; Nikon Corporation).

### miRNA Extraction and Sequencing

Total RNA of EVs from the NG-podo, MA-podo, and HG-podo groups (three replicates/group) were extracted using MagZol reagent (Magen). Library preparation, deep sequencing, and data analysis were conducted by RiboBio Co., Ltd. (Guangzhou, China). Briefly, total RNA was fractionated by gel electrophoresis and small RNAs ranging in length from approximately 18 to 40 nucleotides was used for library preparation. Sequencing was performed using an Illumina HiSeqTM 2500 instrument (Illumina). miRNA read counts were normalized using the trimmed mean of M-values normalization method and differential expression analysis was performed using edgeR package. Differentially expressed miRNAs between two groups were identified *via* |log_2_(fold change)| > 1 and probability *p* < 0.05. Profiling of miRNA expression among samples was conducted by bidirectional hierarchical cluster analysis.

### Validation of miRNAs by Quantitative Reverse Transcription-Polymerase Chain Reaction

The differentially expressed miRNAs were validated by quantitative reverse transcription-PCR (RT-qPCR). EV samples were spiked with 1 pmol cel-miR-39-3p (RiboBio Co., Ltd.) after being fully lysed by MagZol. RNA were reverse transcribed for cDNA synthesis using *Evo M-MLV* RT Kit for qPCR (Accurate Biotechnology). qPCR was performed using SYBR Green Premix *Pro Taq HS* qPCR Kit (Accurate Biotechnology) on a CFX96 Real-Time PCR System (Bio-Rad). The relative expression of miRNAs was calculated by 2^−ΔΔCt^ method with cel-miR-39-3p as an external reference. All the bulge-loop miRNAs RT primers and qPCR primers were purchased from RiboBio Co., Ltd. (Guangzhou, China).

### Target Gene Prediction and Functional/Pathway Enrichment Analysis

The online databases TargetScan,^1^ miRDB,^2^ and miRWalk^3^ were used to predict differentially expressed miRNAs. To improve the accuracy of prediction, only genes that were predicted in all three databases were selected as target genes for further analysis. The common genes were visualized using the online tool Draw Venn Diagram.^4^ Then, the online database DAVID (Database for Annotation Visualization and Integrated Discovery) was used for Gene Ontology (GO) enrichment analysis and Kyoto Encyclopedia of Genes and Genomes (KEGG) pathway enrichment analysis based on predicted target genes.^5^ A value of *p* < 0.05 was considered statistically significant.

### Statistical Analysis

The results are presented as the mean ± standard error of the mean. Statistical analysis was performed using IBM SPSS Statistics for Windows, version 22.0 (IBM). The Student’s *t*-test was used for comparisons between two groups. One-way analysis of variance and the least significant difference test were used for multiple comparisons among the groups. A value of *p* < 0.05 was considered statistically significant.

## Results

### EV Characterization

**Figure 1:**
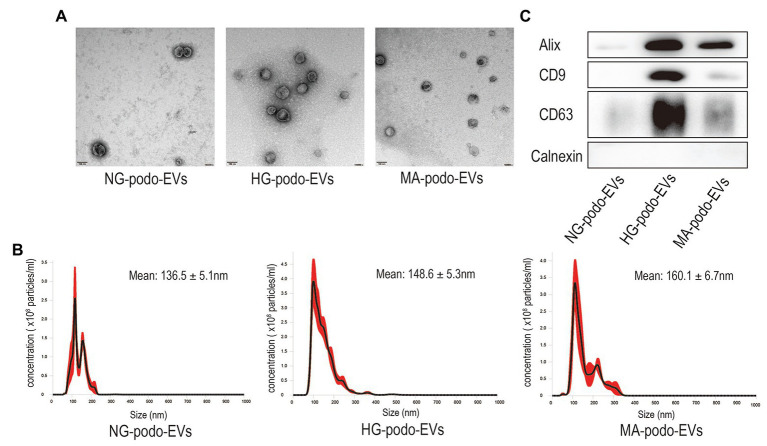
Characterization of EVs isolated from podocytes after NG, MA, and HG treatment. **(A)** EVs were observed by TEM. Magnification, ×100,000. Scale bar, 100 nm. **(B)** The size distribution of EVs was determined by NTA. **(C)** Western blot analysis of Alix, CD9, and CD63, and the EV negative marker Calnexin. HG, high glucose; MA, mannitol; NG, normal glucose; podo, podocytes; NTA, nanoparticle tracking analysis; TEM, transmission electron microscopy.

TEM, NTA, and western blot analysis were used to confirm the presence of EVs (Théry et al., 2018). EVs were extracted from the cell culture supernatants of approximately 3.6 × 10^6^ podocytes that treated with NG, MA, and HG stimulation. The TEM images presented in Figure 1A show that the membrane-bound vesicles were round with typical sizes ranging from 50 to 200 nm. As shown in Figure 1B, the results of NTA revealed a broad distribution of vesicle sizes with a mean diameter of approximately 150 nm. Moreover, western blot analysis confirmed the presence of the EV markers Alix, CD9, and CD63, as well as the absence of Calnexin, which is a protein marker related to the endoplasmic reticulum (Figure 1C). The results of western blotting indicated that podocytes secret more EVs when exposed to HG stimulation, as compared to MA and NG treatment. The above results revealed the typical morphology, size distribution, and protein markers of EVs, demonstrating that EVs were successfully extracted from the podocyte culture supernatants.

### Podocytes EVs Were Taken up by PTECs

**Figure 2:**
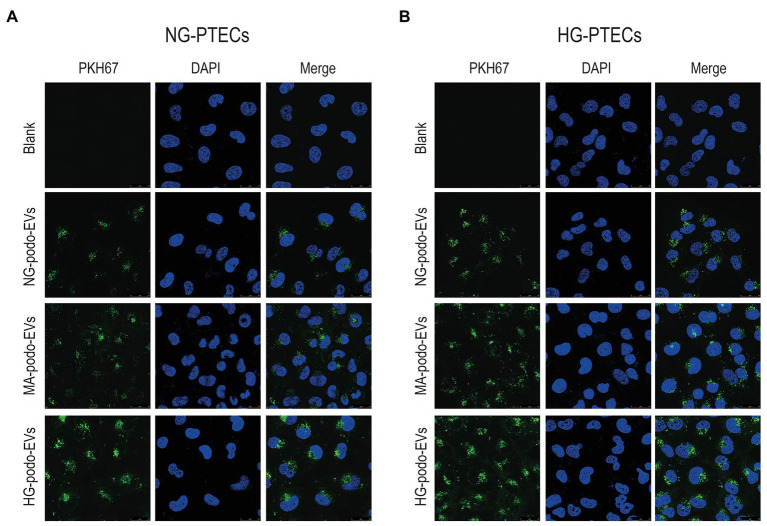
EVs derived from NG/MA/HG-treated podocytes were internalized by cultured PTECs. EVs were labeled with PKH67. Scale bar, 25 μm. **(A)** EVs from podocytes were added to NG-treated PTECs. **(B)** EVs from podocytes were added to HG-treated PTECs. HG, high glucose; MA, mannitol; NG, normal glucose; podo, podocytes; PTECs, proximal tubular epithelial cells.

EVs derived from the same number of podocytes were labeled with the green lipophilic fluorescent dye PKH67 and co-cultured with PTECs to determine whether PTECs could take-up EVs from podocytes. PTECs were treated with normal glucose (NG-PTECs) or high glucose (HG-PTECs). After 48 h of incubation, the cells were fixed and then observed under a confocal laser microscope. As shown in Figure 2, green signals in the cytoplasm of the NG-PTECs and HG-PTECs, indicated that PKH67-labeled EVs were taken up. Moreover, the PTECs internalized more fluorescent EVs when co-cultured with EVs from HG-treated podocytes.

### EVs From HG-Treated Podocytes Induced Apoptosis of PTECs

**Figure 3:**
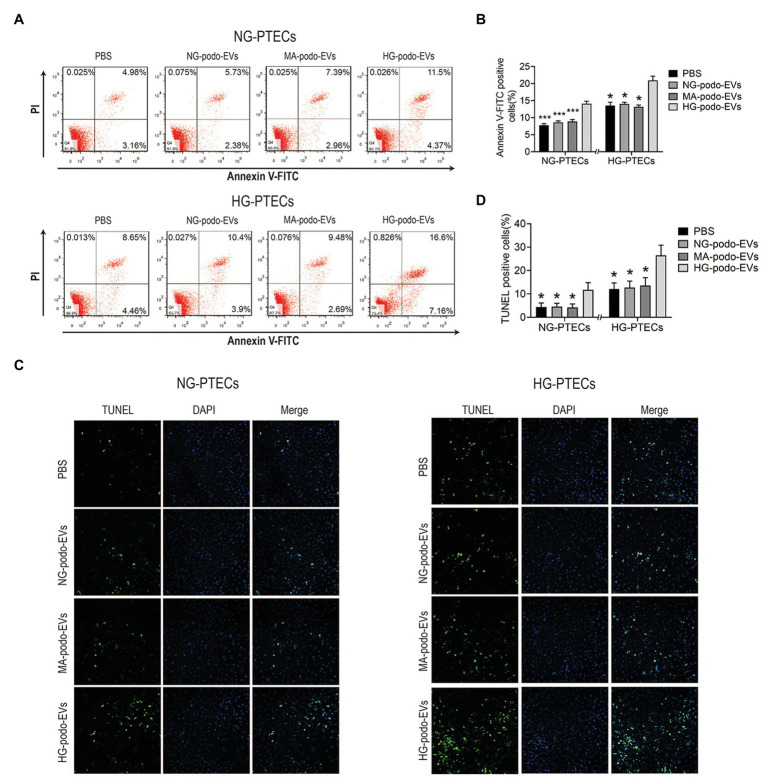
EVs from HG-treated podocytes induced apoptosis of PTECs. Podocytes EVs were incubated with NG/HG-treated PTECs, and the proportion of apoptotic cells was determined by flow cytometry and TUNEL staining. **(A)** Apoptosis was examined using flow cytometry. **(B)** Quantification of Annexin V-FITC positive cells (*n* = 4). **(C)** Apoptosis was detected using TUNEL staining. **(D)** Quantification of TUNEL positive cells. ^*^p < 0.05 vs. HG-podo-EVs group; ^***^p < 0.001 vs. HG-podo-EVs group. HG, high glucose; MA, mannitol; NG, normal glucose; podo, podocytes; PTECs, proximal tubular epithelial cells.

To determine whether EVs from HG-treated podocytes play a pro-apoptotic role in PTECs, cultured PTECs were treated with 30 μg/ml EVs. EVs derived from NG/MA/HG-treated podocytes were added to the cultured NG-PTECs and HG-PTECs. After 48 h of continuous treatment, flow cytometry was performed to measure the proportions of apoptotic PTECs. As shown in Figures 3A,B, the number of apoptotic NG-PTECs (Annexin V-FITC positive) was increased by treatment with EVs from HG-treated podocytes (14.11 ± 0.71%) as compared to NG-PTECs treated with PBS (14.11 ± 0.71% vs. 7.73 ± 0.50%, respectively, *p* < 0.001) and NG-PTECs treated with EVs from NG-treated podocytes (14.11 ± 0.71% vs. 8.62 ± 0.43%, respectively, *p* < 0.001) or MA-treated podocytes (14.11 ± 0.71% vs. 8.87 ± 0.58%, respectively, *p* < 0.001). Similarly, the number of apoptotic HG-PTECs was also increased in the HG-podo-EVs group (20.92 ± 1.27%) as compared to the PBS group (20.92 ± 1.27% vs. 13.56 ± 0.96%, respectively, *p* < 0.05), NG-podo-EVs group (20.92 ± 1.27% vs. 14.08 ± 0.44%, respectively, *p* < 0.05), and MA-podo-EVs group (20.92 ± 1.27% vs. 13.19 ± 1.06%, respectively, *p* < 0.05; Figures 3A,B).

Apoptosis was further confirmed by TUNEL assays. Representative images showed that TUNEL positive cells (recognized as apoptotic cells) were increased after treatment with EVs from HG-treated podocytes (Figure 3C). The percentage of TUNEL positive PTECs were higher in HG-podo-EVs group as compared to other groups (Figure 3D). Taken together, these findings indicate that EVs from HG-treated podocytes exert a pro-apoptotic effect in PTECs cultured under NG or HG conditions.

### Differential Expression of EV miRNA

**Figure 4:**
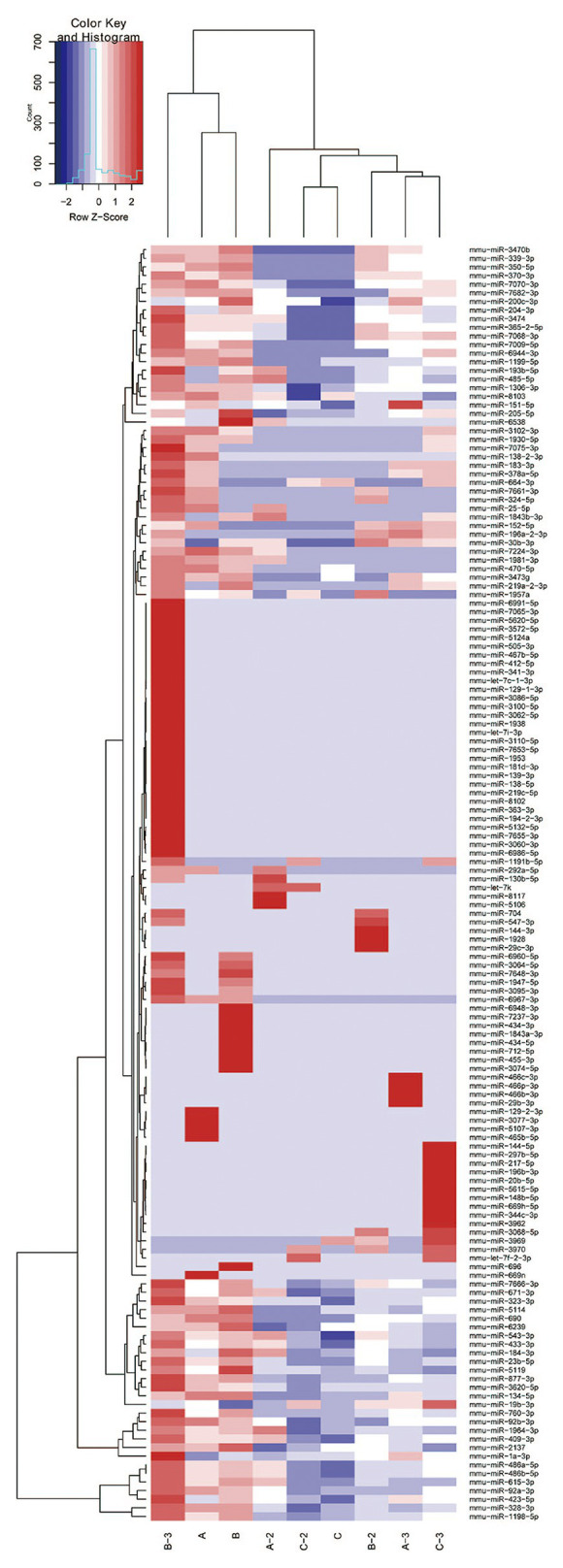
Bidirectional hierarchical cluster result of the differentially expressed miRNAs in EVs derived from NG/MA/HG-treated podocytes (*n* = 3 per group). High expression of miRNAs is shown in red and low expression in blue. A, NG-podo-EVs; B, MA-podo-EVs; C, HG-podo-EVs; HG, high glucose; MA, mannitol; NG, normal glucose.

**Figure 5:**
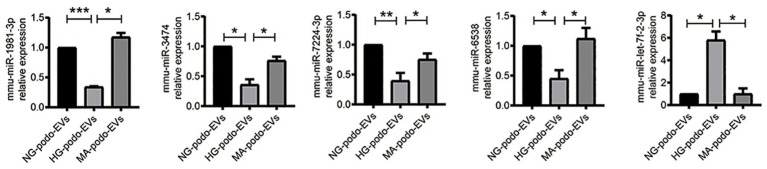
Quantitative reverse transcription-PCR (qRT-PCR) validation of the five miRNAs in podocytes EVs (*n* = 4). ^*^p < 0.05; ^**^p < 0.01; ^***^p < 0.001. HG, high glucose; MA, mannitol; NG, normal glucose; podo, podocytes.

The transfer of EV miRNA is reported to play pathogenic roles in many diseases. To explore the miRNA signature of podocytes EVs and identify differentially expressed miRNAs in response to HG condition, sequencing small RNAs of EVs was performed. The results of bidirectional hierarchical clustering of the samples are presented in Figure 4. A total of 1,915 known miRNAs were identified from all samples after sequencing. A comparison of the HG and NG groups showed that 11 miRNAs were differentially expressed (Table 1), while a comparison of the HG and MA groups showed that 18 miRNAs were differentially expressed (Table 2). To identify unique miRNAs that were differentially expressed in response to HG stimulation, miRNAs that exhibited differential expression in the HG group, as compared to both the MA and NG groups, were selected. The results showed that there were no differences in the expression patterns of the selected miRNAs between the MA and NG groups. Of the five miRNAs selected, the expression levels of mmu-miR-1981-3p, mmu-miR-3474, mmu-miR-7224-3p, and mmu-miR-6538 were downregulated, while that of mmu-let-7f-2-3p was upregulated (Table 3). In addition, to validate the findings obtained from miRNA sequencing, RT-qPCR was performed on the five miRNAs. As the results showed in Figure 5, the expression levels of the five miRNAs in EVs were significantly changed in HG-podo group compared to that of the NG-podo and MA-podo groups. The RT-qPCR results showed similar expression trends and therefore confirmed the sequencing data.

**Table 1:** Differentially expressed miRNAs in EVs from HG-podo group compared to NG-podo group.

miRNA ID	Log_2_(fold change)	*p*
mmu-miR-3969	5.7669	<0.01
mmu-miR-3970	5.6343	<0.01
mmu-miR-25-5p	−5.7129	<0.01
mmu-miR-7224-3p	−5.8863	<0.01
mmu-miR-1981-3p	−5.4928	<0.01
mmu-miR-292a-5p	−5.1123	<0.05
mmu-let-7f-2-3p	4.9189	<0.05
mmu-miR-1191b-5p	4.8885	<0.05
mmu-miR-669n	−6.9665	<0.05
mmu-miR-3474	−2.5186	<0.05
mmu-miR-6538	−5.7653	<0.05

**Table 2:** Differentially expressed miRNAs in EVs from HG-podo group compared to MA-podo group.

miRNA ID	Log_2_(fold change)	*p*
mmu-miR-6538	−8.9623	<0.001
mmu-miR-7224-3p	−5.9352	<0.01
mmu-let-7f-2-3p	4.9189	<0.05
mmu-miR-1981-3p	−5.7412	<0.05
mmu-miR-7661-3p	−5.7562	<0.05
mmu-miR-1947-5p	−5.5333	<0.05
mmu-miR-3095-3p	−5.5295	<0.05
mmu-miR-547-3p	−5.3122	<0.05
mmu-miR-704	−5.3136	<0.05
mmu-miR-7648-3p	−5.2449	<0.05
mmu-miR-6967-3p	−5.2361	<0.05
mmu-miR-324-5p	−5.2071	<0.05
mmu-miR-1a-3p	−3.646	<0.05
mmu-miR-6960-5p	−5.06	<0.05
mmu-miR-3064-5p	−4.934	<0.05
mmu-miR-3474	−3.341	<0.05
mmu-miR-696	−5.9605	<0.05
mmu-miR-204-3p	−3.0463	<0.05

**Table 3:** Differentially expressed miRNAs in podocytes EVs in response to HG.

miRNA ID	Upregulated/downregulated
mmu-miR-1981-3p	Down
mmu-miR-3474	Down
mmu-miR-7224-3p	Down
mmu-miR-6538	Down
mmu-let-7f-2-3p	Up

### Target Gene Prediction

**Figure 6:**
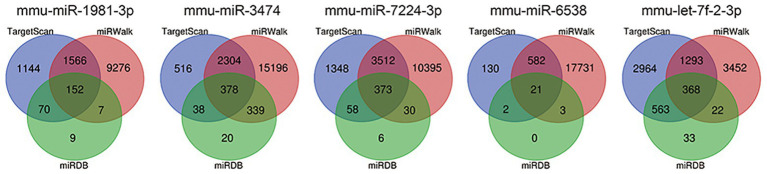
Prediction of target genes of the five miRNAs. Target gene prediction was performed using the online databases TargetScan (blue), miRWalk (red), and miRDB (green). Genes predicted in all three databases (overlapping genes) were selected for further bioinformatics analysis.

To investigate the targets of these five differentially expressed miRNAs, target gene prediction for each miRNA was conducted using the online databases TargetScan, miRDB, and miRWalk. Genes identified in all three databases were selected as potential target genes. As shown in Figure 6, 152, 378, 373, 21, and 368 potential target genes of mmu-miR-1981-3p, mmu-miR-3474, mmu-miR-7224-3p, mmu-miR-6538, and mmu-let-7f-2-3p, respectively, were identified.

### Functional and Pathway Enrichment Analysis

**Figure 7:**
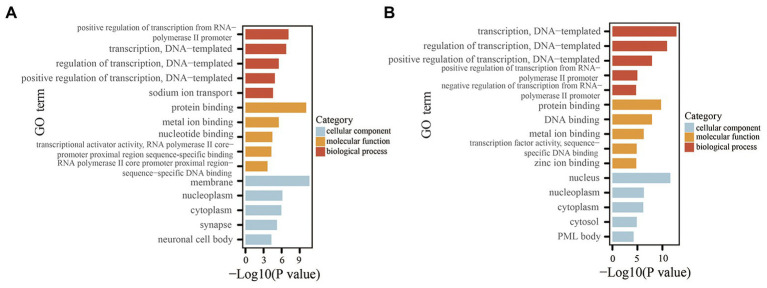
GO analysis of the miRNAs based on the predicted target genes. **(A)** Top five significant GO terms of each category for the downregulated miRNAs. **(B)** Top five significant GO terms of each category for the upregulated miRNA. GO, Gene Ontology.

To explore the potential functions and signaling pathways of the differentially expressed miRNAs, GO enrichment analysis and KEGG pathway enrichment analyses were performed for the differentially expressed miRNAs based on the predicted target genes. GO analysis consisted of three categories: biological process, molecular function, and cellular component. The top five significant GO terms of the three categories are shown in Figure 7. For the downregulated miRNAs, GO analysis was most enriched in “positive regulation of transcription from RNA polymerase II promoter” in the biological process category, “protein binding” in the molecular function category, and “membrane” in the cellular component category (Figure 7A). For the upregulated miRNA (i.e., mmu-let-7f-2-3p), GO analysis was most enriched in “transcription, DNA-templated,” “protein binding,” and “nucleus” in the biological process, molecular function, and cellular component categories, respectively (Figure 7B). The results of KEGG pathway enrichment analysis revealed some known signaling pathways associated with DKD, which included the ErbB, MAPK, Hippo, Wnt, Ras, and PI3K-Akt signaling pathways from the analysis of downregulated miRNAs. KEGG analysis of the upregulated miRNA also identified some DKD-related pathways, including type II diabetes mellitus and the AMPK and HIF-1 signaling pathways. The DKD-related pathways are displayed in Table 4.

**Table 4:** DKD-related pathways from KEGG pathway analysis.

Downregulated/upregulated miRNAs	KEGG ID	Pathway	Count	*p*
Downregulated miRNAs	mmu04012	ErbB signaling pathway	15	<0.001
	mmu04010	MAPK signaling pathway	24	<0.01
	mmu04390	Hippo signaling pathway	16	<0.01
	mmu04310	Wnt signaling pathway	15	<0.01
	mmu04014	Ras signaling pathway	20	<0.01
	mmu04151	PI3K-Akt signaling pathway	26	<0.05
Upregulated miRNA	mmu04930	Type II diabetes mellitus	7	<0.001
	mmu04152	AMPK signaling pathway	7	<0.05
	mmu04066	HIF-1 signaling pathway	6	<0.05

The results of the above experiments showed that EVs derived from HG-treated podocytes induced apoptosis of PTECs, indicating that EVs from HG-treated podocytes could transfer injury information to PTECs. Therefore, based on the enrichment analysis, especially the biological process category (GO analysis), the biological processes and related genes that exert pro-apoptosis effects were identified. The related terms are listed in Table 5. For the downregulated miRNAs (target genes were upregulated), the biological processes associated with promoting apoptosis or injury including “positive regulation of apoptotic process,” “positive regulation of neuron apoptotic process,” and “positive regulation of apoptotic signaling pathway.” For the upregulated miRNA (i.e., mmu-let-7f-2-3p), the target genes were downregulated and the biological processes associated with promoting apoptosis included “negative regulation of extrinsic apoptotic signaling pathway,” and “negative regulation of neuron apoptotic process.” These biological processes and the related genes may provide informative insights for future explorations of the molecular mechanism of tubule apoptosis and injury in DKD.

**Table 5:** Pro-apoptosis related biological processes from GO analysis.

Downregulated/upregulated miRNAs	GO ID	Term	Count	*p*	Genes
Downregulated miRNAs	0043065	Positive regulation of apoptotic process	28	<0.01	ING5, ERBB4, WT1, TGFB2, PYCARD, IL1B, TRP53INP1, UNC5C, PHLDA1, NET1, KCNMA1, EEF1A2, STK4, ATF6, TRIM35, TNFSF10, ITGA6, RPS6KA2, RIPK1, GSK3B, CASP12, SMPD1, LRP6, MAPK8, TRP73, ABL1, BIN1, and SLC9A1
	0043525	Positive regulation of neuron apoptotic process	10	<0.01	KCNMA1, NCF2, GRIK2, GSK3B, IL1B, TFAP2A, MAPK8, ABL1, TGFB2, and ATF2
	2001235	Positive regulation of apoptotic signaling pathway	7	<0.01	ING5, TRP63, TRP53INP1, MAPK8, CAMK2B, CTSC, and TRP73
Upregulated miRNA	2001237	Negative regulation of extrinsic apoptotic signaling pathway	5	<0.01	SH3RF1, ITGA6, RB1CC1, TCF7L2, and ACVR1
	0043524	Negative regulation of neuron apoptotic process	8	<0.05	HIF1A, SET, KDM2B, FYN, GABRB2, MECP2, PPT1, and SIX4

## Discussion

DKD is a serious and common complication of DM and has become one of the most important health concerns worldwide (Tuttle et al., 2014). Glomerular sclerosis and tubulointerstitial fibrosis are major pathologic characteristics of DKD (Tervaert et al., 2010). Proteinuria is a well-known factor that links podocyte damage to tubulointerstitial injury, which triggers tubulointerstitial inflammation and fibrogenesis, and accelerates the decline of renal function (Wang et al., 2000; Abbate et al., 2006; Gorriz and Martinez-Castelao, 2012). However, EVs might be an alternative mechanism that mediate the crosstalk between podocytes and the proximal tubules. Podocytes are located adjacent to the proximal tubules, which represent a likely site for the interactions of podocytes EVs (Lv et al., 2019). Also, EVs serve as excellent carriers of bioactive molecules, since the membrane-bound structure can protect EV contents from degradation during delivery. Thus, EVs from podocytes may travel through the urinary tract and exert various biological effects on PTECs. In the present study, EVs isolated from podocytes culture supernatants were incorporated by HG‐ and NG-treated PTECs. Also, EVs from HG-treated podocytes induced apoptosis of PTECs.

Internephron crosstalk and interactions between the resident cells play important roles in the development of DKD (Qian et al., 2008; Maezawa et al., 2015; Lv et al., 2019). EVs have emerged as vectors for intercellular communication by transferring information between resident cells and participate in the progression of DKD (Wu et al., 2016, 2017; Wang et al., 2018; Zhu et al., 2019). EVs are membrane-bound vesicles that are continuously secreted by cells and facilitate cellular communication by transferring functional cargo consisting of proteins, lipids, DNA, and RNA (EL Andaloussi et al., 2013). The cargo of EVs also plays indispensable roles in many physiological processes, especially the immune response (Bobrie et al., 2011) and tissue regeneration (Krämer-Albers et al., 2007). However, under pathological conditions, such as hypoxia (King et al., 2012), irradiation (Pineda et al., 2019), oxidative stress (Atienzar-Aroca et al., 2016), and infection (Ståhl et al., 2015), the parental cells produce different EVs. The altered quantity and components of the secreted EVs can subsequently mediate abnormal interactions between cells and promote disease progression. In fact, the results of the present study demonstrated that HG conditions induced podocytes to release more EVs as compared to NG and iso-osmolality environments. Considering the important roles of miRNA in the pathogenesis of DKD (Kato and Natarajan, 2015; Lu et al., 2017), miRNA sequencing of podocytes EVs was performed. The results identified 11 differentially expressed miRNAs in response to HG conditions as compared to NG conditions and 18 differentially expressed miRNAs in response to HG conditions as compared to iso-osmolality conditions. In addition, five miRNAs were specifically altered in response to HG stimulation, among which, mmu-miR-1981-3p, mmu-miR-3474, mmu-miR-7224-3p, and mmu-miR-6538 were downregulated, and mmu-let-7f-2-3p was upregulated. These data indicate that podocytes can secret more EVs with altered components under HG conditions and promote apoptosis of PTECs.

In order to determine the biological functions of these five miRNAs, GO and KEGG pathway analyses of the downregulated and upregulated miRNAs were performed based on the predicted target genes. The results of GO and KEGG pathway analyses identified biological processes and signaling pathways that are involved in the development of DKD, including ErbB, MAPK, Hippo, Wnt, Ras, and PI3K-Akt signaling pathways, as determined by analysis of the downregulated miRNAs, and type II diabetes mellitus, and the AMPK and HIF-1 signaling pathways by analysis of the upregulated miRNA (Ni et al., 2015; Chen and Harris, 2016; Persson and Palm, 2017). The GO results also identified enriched biological processes related to apoptosis regulation for both the downregulated and upregulated miRNAs, consistent with our finding that EVs secreted from HG-treated podocytes induce apoptosis of PTECs. The results of bioinformatics analysis provide useful information for future exploration of the molecular mechanisms underlying tubule apoptosis and injury in DKD. However, whether these pro-apoptosis effects are mediated by EV miRNA was not demonstrated in this study, thus further experiments are needed to confirm these findings. Moreover, due to the diversity and complexity of EV components, other bioactive molecules, such as proteins and non-coding RNAs, should also be considered in the investigation of EV-mediated crosstalk between podocytes and PTECs.

To the best of our knowledge, this is the first study to investigate the effects of HG-treated podocytes EVs on PTECs and the first to examine miRNA profiles in EVs from HG-treated podocytes. However, there were some limitations to this study. First, the sample sizes of the groups for miRNA sequencing were relatively small. Thus, larger samples sizes are necessary to confirm the sequencing results. Second, the findings of bioinformatics analysis were based on in silico analysis. Therefore, the bioinformatics findings must be further validated. Finally, further experiments are essential to elucidate the role of EV miRNA from HG-treated podocytes in the induction of apoptosis of PTECs.

In conclusion, the results of the present study demonstrated that EVs derived from HG-treated podocytes can induce apoptosis of PTECs. Here, five differentially expressed miRNAs in EVs from HG-treated podocytes were identified. These findings provide new insights into the pathogenesis of DKD.

## Data Availability Statement

Publicly available datasets were analyzed in this study. This data can be found here: the NCBI Gene Expression Omnibus (GSE154284).

## Author Contributions

YH, RL, XY, and XL designed the study and wrote the manuscript. LZ, YC, WD, and XZ performed the experiments. HY, SZ, ZX, and ZY performed the bioinformatics analysis. WW, CL, ZL, and SL analyzed and interpreted the data. ZD revised the manuscript. All authors contributed to the article and approved the submitted version.

### Conflict of Interest

The authors declare that the research was conducted in the absence of any commercial or financial relationships that could be construed as a potential conflict of interest.

## Footnotes

^1^http://www.targetscan.org/

^2^http://www.mirdb.org/

^3^http://mirwalk.umm.uni-heidelberg.de/

^4^http://bioinformatics.psb.ugent.be/webtools/Venn/

^5^https://david.ncifcrf.gov/
